# *Borrelia hispanica* Relapsing Fever, Morocco

**DOI:** 10.3201/eid1510.090403

**Published:** 2009-10

**Authors:** M’hammed Sarih, Martine Garnier, Najma Boudebouch, Ali Bouattour, Abdelaziz Rihani, Mohammed Hassar, Lise Gern, Danièle Postic, Muriel Cornet

**Affiliations:** Institut Pasteur, Casablanca, Morocco (M. Sarih, N. Boudebouch, M. Hassar); Institut Pasteur, Paris, France (M. Garnier, D. Postic, M. Cornet); Institut Pasteur, Tunis, Tunisia (A. Bouattour); Idrissi Hospital, Kenitra, Morocco (A. Rihani); University of Neuchâtel, Neuchâtel, Switzerland (L. Gern); Paris Descartes University, Paris (M. Cornet)

**Keywords:** Relapsing fever, Borrelia hispanica, molecular diagnosis, bacteria, vector-borne infections, Morocco, dispatch

## Abstract

We found that 20.5% of patients with an unexplained fever in northwestern Morocco had tick-borne relapsing fever. Molecular detection specific for the 16S rRNA gene identified *Borrelia hispanica*. The noncoding intergenic spacer sequence domain showed high sensitivity and good resolution for this species.

Tick-borne relapsing fever (TBRF) is caused by *Borrelia* species transmitted to humans by infected ticks. This condition is frequently undiagnosed and its true incidence is underestimated (*1*,*2*). TBRF is endemic to sub-Saharan Africa, and the most prevalent *Borrelia* species in this region are *B*. *duttonii* in the eastern region and *B*. *crocidurae* in the western region (*3*–*5*). The disease is rarely detected in northern Africa and Mediterranean countries (*3*,*6*,*7*). *B*. *hispanica* and *B*. *crocidurae* infections have been detected in northern and southern Morocco, respectively, along with the tick vectors responsible for their transmission (*Ornithodoros erraticus* and *O*. *sonrai*, respectively) (*8*). However, local transmission has not been detected in Morocco since the reports of Baltazard et al. in 1954 (*9*) and Rodhain in 1976 (*10*), except for 1 traveler who returned from Spain and Morocco in 2005 with a *B*. *hispanica* infection (*7*).

Conventional diagnosis of TBRF is based on detection of spirochetes in blood smears sampled during the acute febrile phase. However, molecular methods have been shown to be more reliable for diagnosis (*6*,*7*,*11*–*13*). We conducted retrospective and prospective surveys of patients with unexplained fever (suspected TBRF) in northwestern Morocco during 2000–2006 and used 2 genomic regions of *Borrelia* spp. to test blood samples from these patients.

## The Study

**Figure 1:**
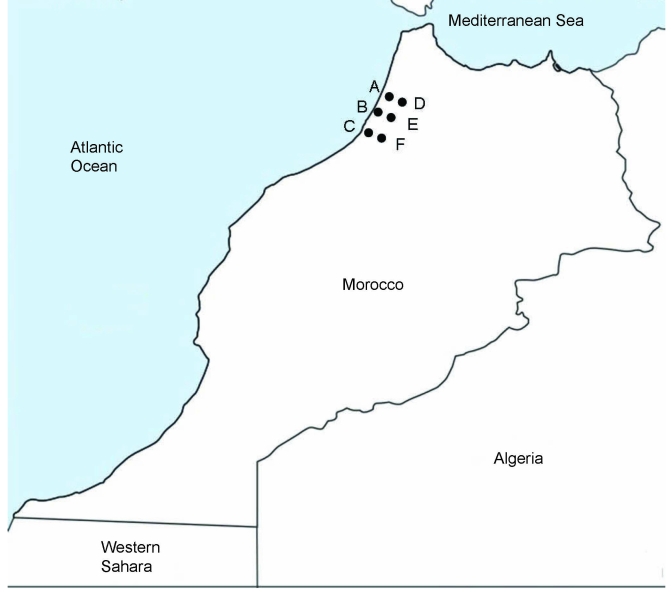
Locations in the Kenitra District of Morocco where tick-borne relapsing fever was diagnosed. A, Sidi Mohamed Lahmar; B, Had Ouled Jelloum; C, Idrissi Kenitra; D, Lalla Mimouna; E, Mnasra; F, Sidi Taybi.

We conducted a preliminary retrospective study during 2000–2004. Results for *Borrelia* spp. screening, which was performed at the same time as diagnostic tests for malaria, were compiled retrospectively from 10 medical centers in the Kenitra District of northwestern Morocco. *Borrelia* spp. spirochetes were identified by observing 200 microscope fields of Giemsa-stained thin blood smears under an oil-immersion objective (magnification ×1,000). Of 75,950 patients, 84 had *Borrelia* spp. infections. On the basis of these results, we conducted active prospective surveillance in 6 medical centers that reported previous TBRF cases (Figure 1).

From January 2005 through December 2006, we investigated all patients with unexplained fever who did not have malaria. All patients were screened for *Borrelia* spp. infection by microscopy and molecular methods in 2005 and only by molecular methods in 2006. Our study design conformed to directives concerning the conduct of clinical trials in Morocco.

DNA was extracted from whole blood sampled during the febrile phase of the patients by using the DNeasy Tissue Kit (QIAGEN, Hilden, Germany). Two PCR methods were used. First, a seminested protocol specific for the entire 16S rRNA gene that used outer primers fD3 and T50 was conducted as described (*11*). The inner primers were REC4, as described (*11*), and RF16SR (5′-pos 867-AGGCGCCACACTTAACACGT-3′-pos 847). REC4 and RF16SR were paired with T50 and fD3, respectively, to obtain 2 amplicons with a 208-bp overlap. These 2 contigs were aligned to obtain the sequence of the entire 16S rRNA gene (1.5 kb). We then conducted a nested protocol specific for the noncoding intergenic spacer region (IGS) as described (*14*). Two negative controls were included in all experiments: a negative extraction control and the PCR mixture without DNA. *B*. *hermsii* DNA was used as a positive control.

PCR products were sequenced on both strands by using Genome Express (Meylan, France) and the same primers as for amplification. All sequences determined in this study were submitted to GenBank (accession nos. FJ827568–FJ827590 for IGS sequences and GQ202254–GQ202265 for 16S rRNA sequences). Multiple sequence alignments were generated with the ClustalW program (www.ebi.ac.uk/Tools/clustalw2/index.html). Phylogenetic analysis was conducted with MEGA software (www.megasoftware.net) as described (*11*).

We included 127 patients in the prospective study. *Borrelia* spp. were detected in 10 (17.5%) of 57 patients in 2005 and 16 (22.9%) of 70 patients in 2006 (mean prevalence rate 20.5%). The patients had not traveled outside Morocco. The most common signs and symptoms associated with fever were chills (88%), myalgia (61%), and gastrointestinal disorders, such as diarrhea and vomiting (54%). Fifteen percent of the patients reported >1 relapse. Patients did not report any tick bites. All patients were successfully treated with doxycycline (100 mg/day for 7 days). Only 2 patients had a diagnosis of TBRF on the basis of microscopy; the diagnosis was confirmed by both PCR methods.

Results for molecular detection are shown in the Table. All blood samples positive according to the 16S rRNA PCR assay were also positive according to the IGS PCR assay. The 26 patients positive for *Borrelia* spp. were from Had Ouled Jelloum and Sidi Mohamed Lahmar; 99 (78%) of the 127 patients were from these 2 sites. At Sidi Mohamed Lahmar, the difference in PCR results for 16S rRNA and IGS remains unexplained. *B*. *hispanica* was identified by BLAST analyses (www.ncbi.nlm.nih.gov/Education/BLASTinfo/information3.html) of the entire 16S rRNA gene sequences from the 12 patients from whom we were able to amplify this gene. Ten of the 12 sequences were identical to the *B*. *hispanica* DQ057988 sequence from GenBank. The 2 remaining sequences (GQ202254 and GQ202257) differed from this sequence by 1 nt (99.92% identity). Levels of similarity between the 12 sequences from Morocco and other relapsing fever species sequences from Africa ranged from 99.4% (2-nt difference) when compared with *B*. *crocidurae* DQ0057990 sequences to 99.35% (7-nt difference) when compared with *B*. *recurrentis* AF107362 sequences.

**Table:** Results of PCR assays for detection of *Borrelia* spp. in 127 patients with unexplained fever in Kenitra, northwestern Morocco

Location	No. patients with positive results/total no. patients (%)	
	16S rRNA seminested PCR	Intergenic spacer sequence nested PCR
Sidi Mohamed Lahmar	2/73 (2.7)	15/73 (20.0)
Had Ouled Jelloum	10/26 (38.5)	11/26 (42.3)
Idrissi Kenitra	0/8	0/8
Lalla Mimouna	0/12	0/12
Mnasra	0/3	0/3
Sidi Taybi	0/5	0/5

All

12/127 (9.4)

26/127 (20.5)

**Figure 2:**
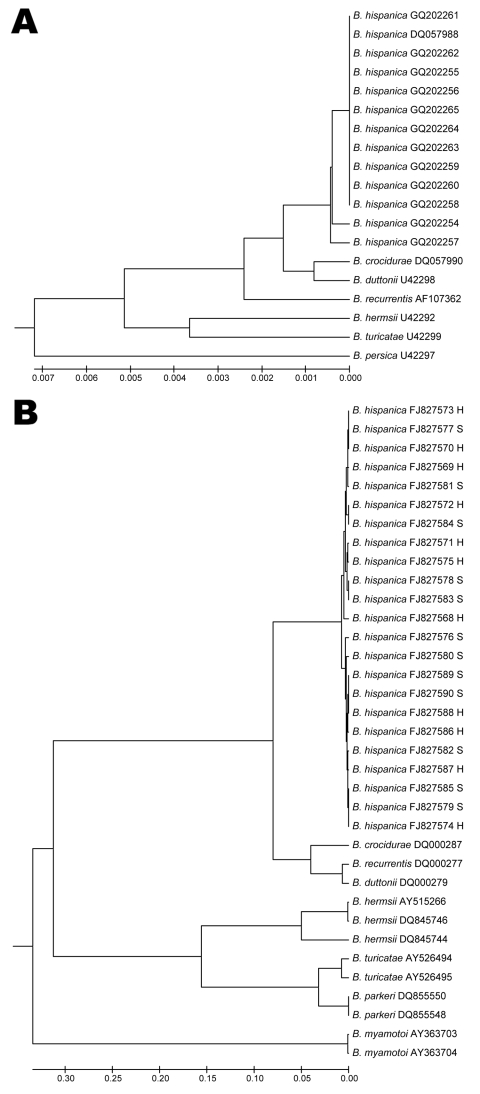
Phylogenetic trees constructed by the unweighted pair group method with arithmetic mean method, by using a pairwise deletion procedure. Distances were calculated by using the Jukes and Cantor method (www.tau.ac.il/~doronadi/jc.pdf). Sequences from GenBank are indicated by accession numbers. A) Phylogenetic tree based on the 16S rRNA sequences of 12 *Borrelia hispanica* DNA samples from patients in Morocco. Sequences from this study submitted to GenBank are accession nos. GQ202254–GQ202265. B) Phylogenetic tree based on noncoding 16S–23S intergenic spacer sequences of 23 *B*. *hispanica* DNA samples from patients in Morocco. Sequences from this study submitted to GenBank are accession nos. FJ827568–FJ827590. The letters S or H indicate the location at which the sample was obtained: S, Sidi Mohamed Lahmar; H, Had Ouled Jelloum. Scale bars indicate genetic distances between DNA sequences.

Phylogenetic analysis of 16S sequences showed single clusters for each relapsing fever species, with small differences between African species, which verified previous results (Figure 2, panel A) (*7*,*15*). Although the IGS locus is not a coding sequence, the level of polymorphism among sequences from Morocco was low. Sequence identity ranged from 99.1% to 100%, with no more than 4-nt differences. We found 6 alleles among 23 IGS sequences. The shallow division between the 2 close branches of the IGS phylogenetic tree did not correspond to the 2 sites at which patients with *B*. *hispanica* relapsing fever were located (Figure 2, panel B).

All IGS sequences obtained from our patients were grouped in a single cluster separate from other *Borrelia* spp. responsible for TBRF. The sequences from Morocco differed from those of other species from Africa, such as *B*. *crocidurae* (77.6% identity with *B*. *crocidurae* DQ000287 sequence) and the *B*. *duttonii*/*B*. *recurrentis* complex (75.5% identity with *B*. *duttonii* DQ000279/*B*. *recurrentis* DQ000277 sequences). Our results verify those of previous studies, which showed that IGS sequences cannot be used to differentiate *B*. *recurrentis* and *B*. *duttonii* from East Africa (*15*). Because the single IGS cluster, which included all sequences from Morocco, included the 12 *B*. *hispanica* identified by their 16S sequences, we conclude that all 23 *Borrelia* spp. DNA samples identified in this study were *B*. *hispanica*.

## Conclusions

The prevalence of *B*. *hispanica* TBRF was high in the Kenitra District of northwestern Morocco. *B*. *hispanica* was detected in 20.5% of patients with unexplained fever. This result may be explained by use of molecular methods for detection, selection of patients with unexplained fever, and living conditions in this region, in which persons live in traditional mud huts and grow groundnuts, particularly at Had Ouled Jelloum, where the highest frequency was observed (*3*,*10*).

Our series highlights the endemicity of TBRF in Morocco, but investigations in other districts are needed. Patients living in Morocco and travelers returning from this country with unexplained fever should be tested for relapsing fever caused by *Borrelia* spp. by using molecular methods.

We have shown that PCR amplification and sequencing of the IGS domain is a sensitive method with a high resolution level for detection of *B*. *hispanica*. This domain may also be useful for detection of other relapsing fever *Borrelia* spp., such as *B*. *crocidurae*, *B*. *hermsii*, *B*. *turicatae*, *B*. *parkeri*, and *B*. *myamotoi*. However, this method is not reliable for detection of *B*. *recurrentis* and *B*. *duttonii*, which cannot be differentiated by their IGS sequences (*15*).
